# Complete Workplace Indoor Smoking Ban and Smoking Behavior among Male Workers and Female Nonsmoking Workers' Husbands: A Pseudo Cohort Study of Japanese Public Workers

**DOI:** 10.1155/2014/303917

**Published:** 2014-03-24

**Authors:** Takahiro Tabuchi, Takahiro Hoshino, Hitomi Hama, Kayo Nakata-Yamada, Yuri Ito, Akiko Ioka, Tomio Nakayama, Isao Miyashiro, Hideaki Tsukuma

**Affiliations:** ^1^Center for Cancer Control and Statistics, Osaka Medical Center for Cancer and Cardiovascular Diseases, 3-3 Nakamichi 1-Chome, Higashinari-ku, Osaka, Osaka 537-8511, Japan; ^2^Graduate School of Economics, Nagoya University, Furo-cho, Chikusa-ku, Nagoya, Aichi 464-8601, Japan

## Abstract

A pseudo cohort study using national cross-sections (2001, 2004, 2007, and 2010) was conducted to examine differences in smoking prevalence under different smoking ban policies such as a complete workplace indoor smoking ban (early or recent implementation) and a partial smoking ban among male public workers and husbands of female nonsmoking public workers. The effectiveness of smoking bans was estimated by difference-in-differences (DID) with age group stratification. The results varied considerably by age and implementation period. Although DID estimates (positive value of DID estimate represents smoking cessation percentage) for both smoking bans on total male smoking were not significant, the over-40 age group indicated a significant DID estimate of 5.0 (95% CI: 0.2, 9.8) for the recent smoking ban. For female workers' husbands' smoking, the over-40 age group indicated positive, but not significant, DID estimates for the early and recent smoking bans of 7.2 (−4.7, 19.2) and 8.4 (−2.0, 18.7), respectively. A complete indoor workplace smoking ban, particularly one recently implemented among public office workers aged over 40, may reduce male workers' smoking and female workers' husbands' smoking compared with a partial smoking ban, but the conclusion remains tentative because of methodological weaknesses in the study.

## 1. Introduction

Tobacco smoking is the most attributable and preventable risk factor for adult mortality and morbidity in Japan [1, 2]. At least ten years of average life expectancy are lost among current smokers in Japan and worldwide [3, 4]. Secondhand tobacco smoke (SHS) is a cause of various illnesses such as neoplastic, respiratory, and cardiovascular diseases [5, 6]. It is estimated that annually at least 4,600 nonsmoking women die from the effects of SHS in Japan [7]. Partly because the health risks of smoking have become generally known, adult smoking prevalence in Japan has declined recently: that is, current smoking has decreased from 48% in 2001 to 33% in 2010 among men and from 14% in 2001 to 10% in 2010 among women [8].

A key intervention in reducing the burden of disease attributable to tobacco use is the smoking ban policy. Along with an increase of population-level knowledge on the risk of SHS, affirmed by the US Surgeon General's Report in 1986, there has been an increase in the number of legislative smoking bans in countries such as Australia, England, and the USA [9]. Smoking bans vary in their comprehensiveness by settings, that is, the extent to which they allow smoking or restrict it to designated areas and where those smoking restrictions occur [9]. In Japan, the Health Promotion Law (HPL) and the Workplace Smoke-free Guideline (WSFG) [10], which promote smoke-free enclosed public places and workplaces, respectively, but allow partial smoking bans as an option, were implemented in 2003 [11], although a partial smoking ban was recommended rather than a complete smoking ban in the WSFG. A partial ban can allow smoking in one part of the same room but not the other and it can also include requirements for smokers and nonsmokers to be separated by a wall and/or different types of ventilation. The partial smoking ban in the WSFG only requires a smoking room where smoke is prevented from leaking into nonsmoking space by a ventilation system which directs exhausted smoke outdoors.

A complete indoor smoking ban has been recommended rather than the partial smoking ban, especially after ratification of the World Health Organization Framework Convention on Tobacco Control (FCTC) by the Japanese government in 2005. The spaces the HPL designated as smoke-free environments include schools, hospitals, gymnasiums, department-stores, restaurants, and public offices, but the execution level of the complete indoor smoking ban differs considerably by setting. For example, the execution rate of the complete indoor smoking ban was 97% in public schools in 2012 [12] but 27% in restaurants in 2011 [13]. Because the law has no penalty for noncompliance, some jurisdictions, such as Kanagawa and Hyogo prefectures, recently implemented their own legislation for public smoking bans which includes penalties [11]. Even in public offices, therefore, there are many varieties in the execution of complete indoor smoking bans by prefectures in Japan. However, because the legislation in Kanagawa and Hyogo cames into operation very recently in April 2010 and April 2013, respectively, we could not evaluate the effect of the legislation on smoking and have focused on the situation before the prefecture-based legislation era in Japan.

Although the main reason for workplace smoking bans is to protect nonsmokers from the harmful health effects of exposure to SHS at work, an incidental impact is to provide a supportive environment for people who want to quit smoking [14]. The diffusion theory [15] suggests that the smoke-free norm of a workplace smoking ban policy can disseminate into adjacent environments such as the home; the workplace smoking ban may therefore affect not only employees but also their families. From the public health perspective, these may provide beneficial impacts [9].

Our objective in this study was to assess the difference in smoking prevalence under different smoking ban policies such as a complete workplace indoor smoking ban and a partial smoking ban among male public workers and husbands of female nonsmoking public workers, before the prefecture-based legislation era in Japan.

## 2. Methods

### 2.1. Study Subjects

We used pseudo cohort data from nationally representative cross-sections which collect information from all household members on health-related factors, such as smoking behavior, every three years: the 2001, 2004, 2007, and 2010 Comprehensive Survey of Living Conditions of People on Health and Welfare, conducted by the Japanese Ministry of Health, Labour and Welfare (MHLW) [16]. To assess the impact of the smoking ban policy in public offices, we used subsamples of male public workers and husbands of female nonsmoking public workers. To interpret the causal inference between a workplace smoking ban and smoking by husbands more easily, husbands who worked at public offices were excluded from the analysis for husbands' smoking. Data were used with permission from MHLW.

### 2.2. Intervention: Smoking Ban in Japanese Governments' Buildings

The execution of a complete indoor smoking ban policy, but no partial smoking ban, in a prefectural government administration building was used as an exogenous proxy indicator for a hypothetical legislative public office smoking ban intervention. To date, previous studies that examined smoking ban legislation and smoking behaviors with consideration of factual execution are scarce [9, 17]. This may be due, in part, to the high level of execution in a number of countries such as Australia, Scotland, and the Netherlands [18–20]. However, it may be appropriate to evaluate smoking ban policy by execution level, as this could reduce underestimation, especially in low execution level countries including Japan [21]. Although Japan implemented HPL and WSFG as well as FCTC for promoting a smoke-free environment, execution of the complete indoor smoking ban remained low, even in government administration buildings, against a target of 100% complete indoor smoking ban.

In 2000, 42 out of 47 prefectural government offices executed partial smoking bans and five allowed smoking in the workplace [22], although we had no specific data for these five prefectures. To investigate the local situation for smoking ban policy across Japan, Yamato and colleagues conducted a mail survey of Japanese local government buildings (including 47 prefectural government offices, 46 prefectural capital municipality offices, 23 wards in Tokyo, and 5 other metropolitan cities) in 2007, 2008, 2010, 2011, and 2013 (response rates were 100% because of intensive reminder notice) [23]. The execution date of the complete indoor smoking ban in governmental building was reported. Of 47 prefectures only 61% executed a complete indoor smoking ban in government office buildings instead of a partial smoking ban in June 2011, although no prefecture allowed smoking in working areas. Based on the implementation period of the complete indoor smoking ban in the building, we classified prefectures into three categories: “Partial smoking ban (reference area)” where smoking was allowed in designated rooms or areas in June 2011; “Early smoking ban” where the prohibition of indoor smoking started between 2003 and 2007 and continued; and “Recent smoking ban” where the ban started between 2008 and 2011. Forty-seven prefectures in Japan were categorized as follows; “Partial smoking ban”: Aomori, Iwate, Fukushima, Gunma, Tokyo, Niigata, Ishikawa, Gifu, Shizuoka, Aichi, Mie, Tottori, Hiroshima, Nagasaki, Kumamoto, Oita, Miyazaki, and Kagoshima; “Early smoking ban”: Yamagata, Ibaragi, Saitama, Kanagawa, Yamanashi, Nagano, Osaka, Hyogo, Yamaguchi, Kochi, Saga, and Okinawa; “Recent smoking ban”: Hokkaido, Miyagi, Akita, Tochigi, Chiba, Toyama, Fukui, Shiga, Kyoto, Nara, Wakayama, Shimane, Okayama, Tokushima, Kagawa, Ehime and Fukuoka. This information was merged with the survey data on the basis of subjects' prefecture of residence. Because the decision regarding the smoke-free policy had been made prior to its execution and had potential impact prior to its implementation (e.g., via anticipation effects) [24], data from May 2003 to May 2011 were used in the study.

### 2.3. Smoking Outcome

The outcome was current smoker prevalence among male employees and husbands of female nonsmoking employees, because there were few female smokers. Current smokers were defined as persons who smoked cigarettes regularly at the time of survey. Smoking behavior was surveyed based on the following four categories: (a) “I do not smoke"; (b) “I smoke every day"; (c) “I smoke occasionally but not every day"; and (d) “I have stopped smoking for more than one month". We categorized (b) and (c) as current smoker. Unfortunately, the information on former smokers (d) was biased [25], potentially because many quitters selected (a) instead of (d); so we could not use this variable to estimate smoking cessation rates. However, current smoker prevalence was reliable because this was consistent with current smoker prevalence in another Japanese representative study of the National Health and Nutrition Survey [25].

### 2.4. Statistical Analysis

**Figure 1:**
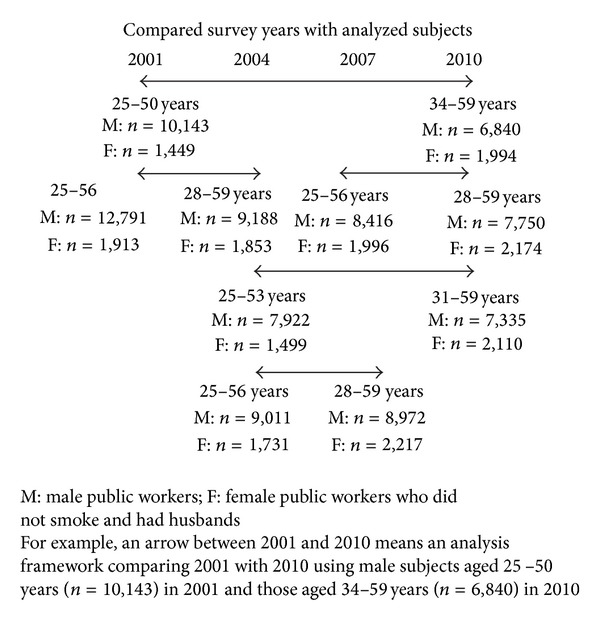
Analytic frameworks with their age groups and total number that were used as study subjects.

Because Japanese people typically retire at 60 years and over, subjects aged 25 years at baseline and up to 59 years in the follow-up period were analyzed by pseudo-cohort methods [26]: for example, persons aged 25–50 years in 2001 were aged 34–59 years in 2010 (Figure 1). Japanese adults aged over 25 years were less likely to start smoking [27], and therefore decreases from pre- to post-current smoker prevalence could be assumed as smoking cessation rates. To evaluate the effect of a complete indoor workplace smoking ban (intervention) on smoking behavioral changes, a difference-in-differences (DID) estimate was calculated by subtracting post (follow-up) outcome rate from pre (baseline) outcome rate in the intervention and reference groups [16, 28]:

(1) $$\begin{array}{c} \text{DID}={\text{difference}}_{\text{intervention}}-{\text{difference}}_{\text{reference}}. \end{array}$$

The positive value of the DID estimate represents the smoking cessation percentage, while the negative value may indicate that of relapse of smoking. Since different tendencies for the health-related behavior between the old and the young were expected [29], we stratified subjects over and under 40 years in baseline with each group representing roughly half of the sample.

Firstly, we compared smoking rates between 2001 and 2010. Next, other time periods such as 2007–2010 were analyzed to validate the result (Figure 1). Furthermore, to compensate for methodological weaknesses which arose from the lack of consideration of background differences between pre- and post-characteristics, confounding factor (such as marital status and housing tenure)-adjusted DIDs were implemented as a sensitivity analysis (see supplementary data). Probability values for statistical tests were two tailed, and *P* < 0.05 was regarded as statistically significant. All statistical analyses were performed using SAS version 9.2 (SAS Institute, Cary, NC).

## 3. Results

Data were available for 247,195 (response rate: 87.3%) households in 2001, 220,836 (79.8%) in 2004, 229,821 (79.9%) in 2007 and 228,864 (79.1%) in 2010. Of these, subsamples of male public workers (*n* = 10, 143–12,791 in 2001, 7,922–9,188 in 2004, 8,416–8,972 in 2007 and 6,840–7,750 in 2010) and husbands of female non-smoking public workers (*n* = 1, 449–1,913 in 2001, 1,499–1,853 in 2004, 1,996–2,217 in 2007 and 1,994–2,174 in 2010) were analyzed (Figure 1). Sample numbers in 2001 and 2010 according to the smoking ban categories and characteristics are shown in Table 1 and supplementary Table S1 and S2 in supplementary materials available online at http://dx.doi.org/10.1155/2014/303917.

Current smoker prevalence, the decrease and DID estimates (effect sizes) among male public office workers according to smoking ban categories are shown in Table 2. Current smoker prevalence decreased from 46.4% in 2001 to 31.6% in 2010 among total male workers. It could be assumed that 14.8% (31.9% of smokers) men stopped smoking during 2001–2010. DID estimates for early and recent smoking bans were not significant among total men: 0.9 (95%CI: −3.0, 4.7) and 1.8 (−1.5, 5.2), respectively. The over 40s age groups indicated significant DID estimates of 5.0 (0.2, 9.8) for the recent smoking ban, although the younger groups did not show significant DID estimates for either smoking ban.

Table 3 shows current smoker prevalence, the decrease and DID estimates among husbands of female nonsmoking public office workers according to smoking ban categories. Spousal (husbands') smoking prevalence decreased from 52.7% in 2001 to 34.9% in 2010 among total female nonsmokers. It could be assumed that 17.8% (33.8% of spousal smokers) husbands stopped smoking during 2001–2010. DID estimates for early and recent smoking bans on spousal smoking was not significant among total female workers. The over 40s age group indicated positive DID estimates for early and recent smoking bans of 7.2 (−4.7, 19.2) and 8.4 (−2.0, 18.7), respectively, although these were not statistically significant.

Table 4 shows DID estimates by several periods before and after 2007, such as 2007–2010, according to smoking ban categories. As for the recent smoking ban, after 2007, the DID estimate among the over 40s was significant for male current smoking and not statistically significant but had a positive value for spousal smoking; 5.7 (1.5, 10.0) and 4.6 (−3.5, 12.7), respectively, although those were nearly zero before 2007. The early smoking ban showed DID estimates for male current smoking were around zero with small range, while those for spousal smoking showed positive values among the over 40s, especially after 2004 including 2004–2010. DID estimates for 2004–2010 were rather higher than those for other time periods for both recent and early smoking bans, particularly among the over 40s; that is, statistically significant results of 5.5 (0.9, 10.1) for recent smoking ban among over 40s male workers, 11.8 (3.2, 20.5) for early smoking ban among husbands of all female workers, 13.6 (2.6, 24.6) for early smoking ban among husbands of over 40s female workers and 11.0 (1.7, 20.2) for recent smoking ban among husbands of over 40s female workers. Furthermore, the sensitivity analysis showed smoking-related factors-adjusted DID results did not largely differ (data not shown).

## 4. Discussion

There is insufficient evidence as to whether a complete smoking ban decreases tobacco use compared with a partial smoking ban [9]. We found that the complete workplace indoor smoking ban, particularly that recently implemented among over 40s public office workers, decreased workers' smoking prevalence compared with a partial ban, especially after 2007. This result of decreased prevalence following a workplace smoking ban is in line with previous studies [14], suggesting a new aspect of the comparison between a complete smoking ban and a partial smoking ban. We also found the workplace smoking ban indicated positive values, although mostly non-significant, on the decrease of husbands' smoking prevalence among over 40s female nonsmoking workers compared with a partial ban. This may imply an increase in smoke-free homes after the implementation of a workplace smoking ban among over 40s female nonsmoking workers. This is in line with previous studies that found smoke-free legislation stimulated the adoption of smoke-free homes [30]. The workplace smoking ban may have a beneficial impact on smoking workers, nonsmoking workers and their families. However, our findings remain tentative because of limited significant results and methodological weaknesses in this study.

In the WSFG in 2003 [10], construction of a comfortable working environment was highlighted rather than workers' health. However, workers' health harm reduction was prioritized in a recent report for workplace smoke-free policy by the MHLW in 2010 [31]. In the context of the new report and the HPL [11] in Japan, all employers have a responsibility and statutory duty to provide and maintain a working environment which is safe and free from risks to health including SHS exposure. Although we only accessed public office workers in the study, generally, the most heavily exposed and most at risk are those working in the hospitality industry such as bar workers, waiters and waitresses [32]. Intake of SHS in bar staff can be four times higher than that arising from living with at least one smoker [33]. The health risks to these employees are therefore especially high, and need to be prevented. Governments initially tend to implement the law affecting only public or unavoidable places [32]. This may widen the degree of inequality in the smoking ban between public and non-public places, although compliance with the law is also important. Thus, from the equity perspective, complete smoking ban policies for all workplaces, including not only public office but also the hospitality industry, must be required.

Unlike the USA where tobacco taxation differs by states, the same cigarette price is applied throughout Japan and there are no media anti-smoking campaigns [11]. Therefore, the impact of these measures which are most influential factors on smoking behavior could be ignored as a strength of this study. Simultaneously, the underlying downward trend in smoking prevalence observed between 2001 and 2010 could be taken into account by the DID method [16].

### 4.1. Workplace Smoking Ban and Husbands' Smoking

A workplace smoking ban may increase awareness of the dangers to nonsmokers of SHS, and help establish norms regarding the inappropriateness of smoking around nonsmokers. The norm of unacceptable smoking around nonsmokers, resulting from compliance with the indoor smoking ban policy, might influence people to adopt such rules voluntarily for their homes [14], and might improve husbands' smoking cessation by enhancing conjugal support and communication [34]. Thus, the mechanism between the workplace smoking ban and home smoking behavior may decrease husbands' smoking.

In a previous review, workplace smoking bans were deemed to have a smaller effect on smoking behavior than home smoking bans in studies that analyzed both workplace and home smoking bans simultaneously [14]. However, according to the above mechanism, voluntary home smoking bans may mediate between workplace smoking bans and smoking behavior. Thus, the adjustment for home smoking bans may result in underestimation of the effect of workplace smoking ban; that is, although the variable of home smoking ban was not used in the current study, it may be appropriate for evaluation on the effect of a workplace smoking ban.

### 4.2. Effect Modifications

In this study, large age group differences in the effect of a smoking ban on smoking behaviors were seen. Generally, smoking cessation may be more difficult for older than for younger adults, because of a longer duration of smoking and thus a stronger nicotine dependence. However, the observed age group difference in the study is not surprising, because older people are more likely to conduct healthy behavior change than younger people [35]. Although few previous studies have examined the smoking ban using age group stratification, a lower effectiveness of smoking restrictions among young populations was observed [36], consistent with this study. Johnson et al. note that older people are more likely than young people to try to avoid unnecessary risks owing to their accumulated experience of health risks over a lifetime [35]. Another reason for the age group differences might be similar to resistance to the smoking ban by adolescents who start smoking as a form of rebellion [37], and thus a positive effect of smoking ban was not observed among the under 40s. Furthermore, different personal compositions, such as age and housing tenure, might cause a difference, although the results of sensitivity analyses adjusting these covariates did not materially differ.

In terms of difference due to implementation period, the effect of recent smoking ban was observed, particularly in 2007–2010, although the effect of the early smoking ban, which was implemented in 2003–2007, was not stable (Table 4). This might be due to a potential interaction. The smoking ban was one component of a multi-component effort to reduce tobacco use. The prefectural execution of the complete indoor smoking ban might occur during a period when other tobacco control strategies were relatively steady in the prefecture. A recent smoking ban might have a better interaction effect with a recently improved environment which promotes smoking cessation than an early smoking ban, because population norms against smoking had been reinforced by recent other tobacco control measures such as increased tobacco taxation and improved cessation assistance [11]. Thus, it is not generally possible to attribute all changes in smoking behavior to the smoking ban.

In terms of husbands' smoking, a wide range of baseline smoking prevalence by stratified categories might result in unstable DID estimates with limited significance. This might be due to chance and small sample size.

### 4.3. Limitations

There are several other limitations in the study. First, smoking outcomes were self-reported without biomarker validation, but the reliability of self-reporting smoking behavior was generally high [38]. Second, because this study is based on repeated cross sections instead of longitudinal data, changes in one individual could not be specified. Therefore, results may be biased by accidental distributions between different years. Longitudinal studies, however, have the problem that disadvantaged people are likely to leave the study. In this study, all respondents with characteristics of disadvantage could be included. Furthermore, the prefecture-based proxy indicator, which was used for intervention identification, may also lead to an ecological fallacy, an accident or underestimation by misclassification. Third, because public workers were studied, the ability to generalize from the results might be limited. The smokers in public offices might be more susceptible to pressure to change their behavior [14]. Therefore, this may lead to overestimation. Fourth, the execution of the smoking ban was not random. For example, Kanagawa prefecture implemented its own legislation to provide a smoke-free environment. In some cases, it has been argued that antismoking sentiments drove the passage of the law and reductions in smoking behaviors. We could not control for antismoking sentiments in the population, although strong leadership on making-decision by local governors was believed to be important for the implementation of legislation in Japan [39].

## 5. Conclusions

We examined whether a workplace complete indoor smoking ban would reduce male workers' smoking and female workers' husbands' smoking, compared with a partial ban, among Japanese public workers. The effectiveness of smoking bans considerably varied by age and period. A complete workplace indoor smoking ban, particularly one recently implemented among public office workers aged over 40, may reduce male workers' smoking and female workers' husbands' smoking compared with a partial ban, although other categories indicated weak, negative or no impact on smoking cessation.

## Supplementary Material

Additional data for the sensitivity analysis, supplementary references and analyzed sample numbers according to basic characteristics or smoking ban categories were indicated in the Supplementary Material.

## Figures and Tables

**Table 1:** Subjects number among public office workers according to smoking ban categories.

Smoking ban categories	Men				Married nonsmoking women			
	2001		2010		2001		2010	
	*N*	%	*N*	%	*N*	%	*N*	%
Total subjects								
Partial smoking ban	3785	37.3	2644	38.7	472	32.6	775	38.9
Early smoking ban (2003–2007)	4001	39.5	2576	37.7	651	44.9	733	36.8
Recent smoking ban (2008–2011)	2357	23.2	1620	23.7	326	22.5	486	24.4
Subjects aged 25–39 years^a^								
Partial smoking ban	1896	38.0	1412	38.8	177	31.1	442	42.0
Early smoking ban (2003–2007)	1952	39.2	1373	37.7	265	46.5	351	33.3
Recent smoking ban (2008–2011)	1137	22.8	855	23.5	128	22.5	260	24.7
Subjects aged 40–50 years^a^								
Partial smoking ban	1889	36.6	1232	38.5	295	33.6	333	35.4
Early smoking ban (2003–2007)	2049	39.7	1203	37.6	386	43.9	382	40.6
Recent smoking ban (2008–2011)	1220	23.7	765	23.9	198	22.5	226	24.0

^a^Categorized by age in June 2001.

Notes: Subjects number in other framework such as 2004 and 2007 was similar with this distribution (data not shown).

**Table 2:** Current smoker prevalence, decrease, and difference-in-differences (DID) estimates among male public office workers according to smoking ban categories.

Smoking ban categories	Current smoker prevalence				Effect size of the public office smoking ban
	2001 %	2010 %	Decrease, % point (95% CI)	Decrease by percent change, %	DID estimates^a^, % point (95% CI)
Total male workers	46.4	31.6	14.8 (13.5, 16.2)	31.9	
Partial smoking ban	46.8	32.9	13.9 (12.6, 15.3)	29.8	
Early smoking ban (2003–2007)	46.8	32.0	14.8 (13.5, 16.1)	31.6	0.9 (−3.0, 4.7)
Recent smoking ban (after 2007)	45.7	30.0	15.8 (14.4, 17.1)	34.5	1.8 (−1.5, 5.2)
Male workers aged 25–39 years^b^					
Partial smoking ban	47.4	33.3	14.1 (12.8, 15.5)	29.8	
Early smoking ban (2003–2007)	47.3	33.2	14.1 (12.8, 15.4)	29.8	0.0 (−5.5, 5.4)
Recent smoking ban (After 2007)	43.6	30.9	12.8 (11.4, 14.1)	29.2	−1.4 (−6.0, 3.3)
Male workers aged 40–50 years^b^					
Partial smoking ban	46.2	32.4	13.8 (12.5, 15.2)	29.9	
Early smoking ban (2003–2007)	46.4	30.7	15.7 (14.3, 17.0)	33.8	1.8 (−3.7, 7.4)
Recent smoking ban (After 2007)	47.7	28.9	18.8 (17.5, 20.1)	39.4	5.0 (0.2, 9.8)

^a^The category of “Partial smoking ban” was used as a reference. Positive value of DID estimates represents smoking cessation rates among male workers.

^b^Categorized by age in June 2001.

CI: confidence interval.

**Table 3:** Current smoker prevalence, decrease, and difference-in-differences (DID) estimates among husbands of female nonsmoking public office workers according to smoking ban categories.

Smoking ban categories	Current smoker prevalence of husbands				Effect size of the public office smoking ban
	2001 %	2010 %	Decrease, % point (95% CI)	Decrease by percent change, %	DID estimates^ a^, % point (95% CI)
Husbands of total female workers	52.7	34.9	17.8 (16.4, 19.2)	33.8	
Partial smoking ban	51.9	35.4	16.6 (15.2, 17.9)	31.9	
Early smoking ban (2003–2007)	47.2	32.3	14.9 (13.6, 16.3)	31.6	−1.6 (−10.5, 7.2)
Recent smoking ban (after 2007)	55.9	36.0	19.9 (18.5, 21.3)	35.6	3.3 (−4.3, 11.0)
Husbands of female workers aged 25–39 years^b^					
Partial smoking ban	58.2	34.4	23.8 (22.5, 25.1)	40.9	
Early smoking ban (2003–2007)	46.9	35.4	11.5 (10.1, 12.8)	24.5	−12.3 (−25.8, 1.1)
Recent smoking ban (After 2007)	59.2	38.5	20.8 (19.4, 22.1)	35.1	−3.0 (−14.6, 8.5)
Husbands of female workers aged 40–50 years^b^					
Partial smoking ban	48.1	36.6	11.5 (10.1, 12.9)	23.9	
Early smoking ban (2003–2007)	47.5	28.8	18.7 (17.4, 20.0)	39.4	7.2 (−4.7, 19.2)
Recent smoking ban (After 2007)	53.6	33.8	19.9 (18.5, 21.2)	37.0	8.4 (−2.0, 18.7)

^a^The category of “Partial smoking ban" was used as a reference. Positive value of DID estimates represents smoking cessation rates among husbands of female workers.

^b^Categorized by age in June 2001.

CI: confidence interval.

**Table 4:** Difference-in-differences (DID) estimates by before and after 2007 time durations, according to smoking ban categories.

Smoking ban categories	DID estimates^a^				
	Before 2007		After 2007	Before and after 2007	
	2001–2004	2004–2007	2007–2010	2001–2010^b^	2004–2010
	% point (95% CI)	% point (95% CI)	% point (95% CI)	% point (95% CI)	% point (95% CI)
Total male workers					
Early smoking ban (2003–2007)	0.8 (−2.7, 4.3)	−1.6 (−5.3, 2.1)	1.0 (−3.0, 5.1)	0.9 (−3.0, 4.7)	−1.1 (−5.1, 2.9)
Recent smoking ban (after 2007)	0.6 (−2.5, 3.6)	−0.8 (−4.0, 2.5)	3.2 (−0.4, 6.7)	1.8 (−1.5, 5.2)	2.2 (−1.3, 5.7)
Male workers aged 25–39 years^c^					
Early smoking ban (2003–2007)	3.9 (−1.6, 9.4)	−3.8 (−9.8, 2.1)	−3.0 (−10.5, 4.5)	0.0 (−5.5, 5.4)	−6.8 (−12.9, −0.8)
Recent smoking ban (after 2007)	−1.7 (−6.5, 3.1)	−0.8 (−6.2, 4.5)	−2.5 (−9.1, 4.0)	−1.4 (−6.0, 3.3)	−2.3 (−7.6, 3.0)
Male workers aged 40^c^–59^d^ years					
Early smoking ban (2003–2007)	−1.4 (−5.9, 3.2)	−0.6 (−5.3, 4.2)	2.8 (−2.0, 7.6)	1.8 (−3.7, 7.4)	3.2 (−2.1, 8.5)
Recent smoking ban (after 2007)	2.0 (−2.0, 5.9)	−1.2 (−5.4, 3.0)	**5.7 (1.5, 10.0)**	**5.0 (0.2, 9.8)**	**5.5 (0.9, 10.1)**
Husbands of total female workers					
Early smoking ban (2003–2007)	−8.4 (−17.1, 0.3)	6.4 (−2.0, 14.7)	2.1 (−5.8, 10.0)	−1.6 (−10.5, 7.2)	**11.8 (3.2, 20.5)**
Recent smoking ban (after 2007)	1.5 (−5.7, 8.8)	1.5 (−5.5, 8.5)	1.6 (−5.4, 8.6)	3.3 (−4.3, 11.0)	4.2 (−3.2, 11.6)
Husbands of female workers aged 25–39 years^c^				
Early smoking ban (2003–2007)	−15.2 (−31.0, 0.5)	4.6 (−10.6, 19.8)	0.0 (−14.6, 14.7)	−12.3 (−25.8, 1.1)	8.7 (−5.9, 23.2)
Recent smoking ban (after 2007)	1.0 (−12.1, 14.0)	−1.3 (−14.5, 11.9)	−6.8 (−20.4, 6.8)	−3.0 (−14.6, 8.5)	−8.7 (−21.4, 3.9)
Husbands of female workers aged 40^c^–59^d^ years					
Early smoking ban (2003–2007)	−6.0 (−16.4, 4.5)	8.1 (−2.0, 18.2)	2.8 (−6.6, 12.2)	7.2 (−4.7, 19.2)	**13.6 (2.6, 24.6)**
Recent smoking ban (after 2007)	1.0 (−7.7, 9.7)	2.0 (−6.4, 10.3)	4.6 (−3.5, 12.7)	8.4 (−2.0, 18.7)	**11.0 (1.7, 20.2)**

^a^The category of “Partial smoking ban" was used as a reference. Positive value of DID estimates represents smoking cessation rates.

^b^Represented from Tables 2 and 3.

^c^Age in baseline period.

^d^Age in follow-up period.

CI: confidence interval.
