# Supplementary material for: Complete Workplace Indoor Smoking Ban and Smoking Behavior among Male Workers and Female Nonsmoking Workers' Husbands: A Pseudo Cohort Study of Japanese Public Workers
Source: Biomed Res Int. 2014 Mar 24;2014:303917. doi: 10.1155/2014/303917 (PMC3982281; doi:10.1155/2014/303917)
Supplement: Supplementary file 1 — Additional data for the sensitivity analysis, supplementary references and analyzed sample numbers according to basic characteristics or smoking ban categories were indicated in the Supplementary Material. [file 303917.f1.docx]

**Supplementary data (online only)**

**Title**

Complete workplace indoor smoking ban and smoking behavior among male workers and female non-smoking workers' husbands: A pseudo-cohort study of Japanese public worker

**Supplementary methods**

***Sensitivity analysis***

The impact of the intervention on smoking outcomes was estimated by unadjusted difference-in-differences (DID). However, crude comparisons of pre- and post-outcomes may be contaminated by the effect of biased characteristics that differ between the two groups. Thus, we applied the confounding factors-adjusted DID [1, 2] as a sensitivity analysis. Factors related to smoking behavior were used to present characteristics of study subjects and to control for their possible confounding effects. In line with previous studies [3-5], we used (i) age group, (ii) marital status (married, never married or widowed/divorced), (iii) housing tenure (yes/no), (iv) equivalent household expenditure in a month (tertile) and (v) living in metropolitan areas (yes/no).

**Supplementary results**

Analyzed sample numbers (period 2001-2010) according to basic characteristics are shown in supplementary Table S1. Nationally representative 16,983 male public employees (10,143 in 2001 and 6,840 in 2010) and 3,443 married non-smoking female public employees (1,449 in 2001 and 1,994 in 2010) were analyzed. The figures in 2001-2010 were slightly smaller than sample size of other periods such as 2007-2010 (data not shown), because the usable age range (age of 25 years in baseline and up to 59 years in follow-up period) was narrower in 2001-2010 than in other periods such as 2007-2010. Basic characteristics according to smoking ban categories are shown in Table S2. There was no large difference in distribution of age group, marital status or metropolitan area by smoking ban category. On the other hand, some differences in those of home owner and equivalent household expenditure were observed, but there is no consistent tendency by smoking ban category.

***Sensitivity analysis***

The confounding factors-adjusted DID estimates did not largely differ compared with unadjusted DID estimates in Table 4, although the model complexity in the adjusted DID resulted in a wide confidential interval (data not shown).

**Supplementary references**

[1] A. Abadie A, "Semiparametric Difference-in-Differences Estimators." *Review of Economic Studies,* vol. 72, pp. 1-19, 2005.

[2] T. Tabuchi, T. Hoshino, T. Nakayama et al., "Does removal of out-of-pocket costs for cervical and breast cancer screening work? A quasi-experimental study to evaluate the impact on attendance, attendance inequality and average cost per uptake of a Japanese government intervention." *International Journal of Cancer,* vol. 133, no. 4, pp. 972-983, 2013.

[3] Y. Fukuda, K. Nakamura, T. Takano, "Socioeconomic pattern of smoking in Japan: income inequality and gender and age differences." *Annals of Epidemiology,* vol. 15, no. 5, pp. 365-372, 2005.

[4] Y. Fukuda, K. Nakamura, T. Takano, "Accumulation of health risk behaviours is associated with lower socioeconomic status and women's urban residence: a multilevel analysis in Japan." *BMC public health,* vol. 5, pp. 53, 2005.

[5] M. M. Schaap, A. E. Kunst, "Monitoring of socio-economic inequalities in smoking: learning from the experiences of recent scientific studies." *Public Health,* vol. 123, no. 2, pp. 103-109, 2009.

| Table S1. Subjects number according to basic characteristics | | | | | | | | | | |
| --- | --- | --- | --- | --- | --- | --- | --- | --- | --- | --- |
|  |  | Male workers | | | |  | Married non-smoking female workers | | | |
| Characteristics |  | 2001 | | 2010 | |  | 2001 | | 2010 | |
|  |  | N | % | N | % |  | N | % | N | % |
| Age group in 2001^a^ |  |  |  |  |  |  |  |  |  |  |
| 25-29 |  | 1478 | 14.6 | 1073 | 15.7 |  | 111 | 7.7 | 313 | 15.7 |
| 30-34 |  | 1624 | 16.0 | 1190 | 17.4 |  | 216 | 14.9 | 367 | 18.4 |
| 35-39 |  | 1883 | 18.6 | 1377 | 20.1 |  | 243 | 16.8 | 373 | 18.7 |
| 40-44 |  | 2292 | 22.6 | 1532 | 22.4 |  | 394 | 27.2 | 443 | 22.2 |
| 45-50 |  | 2866 | 28.3 | 1668 | 24.4 |  | 485 | 33.5 | 498 | 25.0 |
| Marital status |  |  |  |  |  |  |  |  |  |  |
| Married |  | 7963 | 78.5 | 6098 | 89.2 |  | 1449 | 100.0 | 1994 | 100.0 |
| Never married |  | 2036 | 20.1 | 588 | 8.6 |  | NA |  | NA |  |
| Widowed/Divorced |  | 144 | 1.4 | 154 | 2.3 |  | NA |  | NA |  |
| Home owner |  |  |  |  |  |  |  |  |  |  |
| No |  | 3331 | 32.8 | 1694 | 24.8 |  | 222 | 15.3 | 254 | 12.7 |
| Yes |  | 6812 | 67.2 | 5146 | 75.2 |  | 1227 | 84.7 | 1740 | 87.3 |
| Equivalent household expenditure |  |  |  |  |  |  |  |  |  |  |
| 1st (lowest) tertile |  | 3261 | 32.2 | 2196 | 32.1 |  | 476 | 32.9 | 641 | 32.2 |
| 2nd tertile |  | 3240 | 31.9 | 2261 | 33.1 |  | 458 | 31.6 | 638 | 32.0 |
| 3rd (highest) tertile |  | 3293 | 32.5 | 2162 | 31.6 |  | 461 | 31.8 | 647 | 32.5 |
| Missing |  | 349 | 3.4 | 221 | 3.2 |  | 54 | 3.7 | 68 | 3.4 |
| Metropolitan areas |  |  |  |  |  |  |  |  |  |  |
| No |  | 9034 | 89.1 | 6099 | 89.2 |  | 1339 | 92.4 | 1819 | 91.2 |
| Yes |  | 1109 | 10.9 | 741 | 10.8 |  | 110 | 7.6 | 175 | 8.8 |
| ^a^Categorized by age in June 2001. |  |  |  |  |  |  |  |  |  |  |
